# Plasmonics Meets Biology through Optics

**DOI:** 10.3390/nano5021022

**Published:** 2015-06-09

**Authors:** Luciano De Sio, Giulio Caracciolo, Ferdinanda Annesi, Tiziana Placido, Daniela Pozzi, Roberto Comparelli, Alfredo Pane, Maria Lucia Curri, Angela Agostiano, Roberto Bartolino

**Affiliations:** 1 Beam Engineering for Advanced Measurements Company, Winter Park, FL 32789, USA; 2 Department of Molecular Medicine, Sapienza University of Rome, Viale Regina Elena 291, 00161 Rome, Italy; E-Mails: giulio.caracciolo@uniroma1.it (G.C.); daniela.pozzi@uniroma1.it (D.P.); 3 CNR-Lab. Licryl, Institute NANOTEC, 87036 Arcavacata di Rende, Italy; E-Mails: ferdinanda.annesi@cnr.it (F.A.); alfredo.pane@fis.unical.it (A.P.); roberto.bartolino@fis.unical.it (R.B.); 4 Department of Chemistry, University of Bari, Via Orabona 4, 70126 Bari, Italy; E-Mails: t.placido@ba.ipcf.cnr.it (T.P.); angela.agostiano@uniba.it (A.A.); 5 CNR-IPCF, National Research Council of Italy, Institute for Physical and Chemical Processes, Bari Division, Via Orabona 4, 70126 Bari, Italy; E-Mails: r.comparelli@ba.ipcf.cnr.it (R.C.); lucia.curri@ba.ipcf.cnr.it (M.L.C.); 6 Department of Physics, University of Calabria, Centre of Excellence for the Study of Innovative Functional Materials, 87036 Arcavacata di Rende, Italy; 7 Interdisciplinary Institute B, Segre of the National Academy dei Lincei, 00165 Rome, Italy

**Keywords:** nanomaterials, plasmonics, DNA, optics

## Abstract

Plasmonic metallic nanoparticles (NPs) represent a relevant class of nanomaterials, which is able to achieve light localization down to nanoscale by exploiting a phenomenon called Localized Plasmon Resonance. In the last few years, NPs have been proposed to trigger DNA release or enhance ablation of diseased tissues, while minimizing damage to healthy tissues. In view of the therapeutic relevance of such plasmonic NPs; a detailed characterization of the electrostatic interaction between positively charged gold nanorods (GNRs) and a negatively charged whole-genome DNA solution is reported. The preparation of the hybrid biosystem has been investigated as a function of DNA concentration by means of ζ-potential; hydrodynamic diameter and gel electrophoresis analysis. The results have pointed out the specific conditions to achieve the most promising GNRs/DNA complex and its photo-thermal properties have been investigated. The overall study allows to envisage the possibility to ingeniously combine plasmonic and biological materials and, thus, enable design and development of an original non invasive all-optical methodology for monitoring photo-induced temperature variation with high sensitivity.

## 1. Introduction

At nanometer scale several domains, such as physics, chemistry, biology, and materials sciences can be found to converge. Plasmonics is a specific area of interest within the realm of nanophotonics and deals with localization and manipulation of light within a nanoscale volume. Nanoparticle plasmonics is a rapidly emerging research field that deals with the fabrication, optical study and application of noble metal nanoparticles (NPs) of various sizes, shapes, and materials [1]. NPs have the unique capability to confine light at the nanoscale trough the excitation of the Localized Plasmonic Resonance (LPR), a phenomenon related to the oscillation of the free electrons localized at the metal (NPs)/dielectric interface. The LPR frequency strongly depends on the composition, size, geometry, dielectric environment and separation distance of NPs [2]. Plasmonic NPs (e.g., gold, silver, *etc*.) have the extraordinary capability to convert external light to heat, as the strong electric field generated around the NPs, due to the LPR effect, is transformed, resulting in nanosized sources of heat [3]. Although challenging to study and measure, the temperature increase at the surface of NPs under optical illumination is an important issue for many applications including gene therapy (GT) [4,5] and nanomedicine [6,7]. To this end, gold NPs (GNPs) have gained a significant role because they are bio-compatible, can be easily functionalized with a large variety of chemical compounds and can be used as nano-carries in GT applications by encapsulating nucleic acid molecules like DNA and RNA [8,9]. The ability of GNPs to interact with and be internalized within cells has fostered research towards their bioconjugation with a variety of biologically relevant compounds and macromolecules [10,11]. In particular, electrostatic interactions between GNPs and DNA molecules has been demonstrated one of the most viable functionalization route to obtain stable GNPs bioconjugates, offering the advantage to control the effective electric charge of the system and, thus, to prevent, or at least minimize, electrostatic repulsion with negatively charged cell membranes. Moreover, GNPs can be photo-heated, due to the LPR mechanism, by irradiation with an external laser source, at a suitable wavelength, able to penetrate biological tissues and to trigger intracellular DNA release or thermal ablation [6,12]. The electrostatic bioconjugation between GNPs and DNA helps to protect the DNA molecules from various enzyme digestions and improves the transfection efficiency; at the same time, the photo-thermal properties of GNPs enable the DNA release process in the target cells. The fabrication of a system bridging a biological moiety with an inorganic nanomaterial impose stringent optical characteristics, as the LPR of NPs needs to be close/fall within a biological window (700–900 nm, first and second biological window, respectively), that is where tissue absorption is low and the penetration depth of the radiation is high [13]. Among different types of metal NPs, gold nanorods (GNRs) present two LPRs, transverse and longitudinal plasmon absorption bands [14]. The latter peak position is widely tunable from the visible to near infrared radiation (NIR) as a function of particle geometry. In this paper, we have investigated the electrostatic bioconjugation between an aqueous solution of positively charged GNRs dispersed in a negatively charged whole-genome DNA solution. A detailed study of the GNRs/DNA complexes has been performed as a function of DNA concentration by means of ζ-potential, dynamic light scattering and gel electrophoresis analysis. The thermal properties of the most promising GNRs/DNA complex have been investigated in terms of localized photo-thermal heating and delocalized temperature variation on the DNA melting, in view of their biomedical application. Compared to previously employed techniques [15,16,17,18], the proposed non-invasive methodology has been found to enable the monitoring of photoinduced temperature variation around GNRs with high sensitivity, thus providing a relevant tool for both biological study and therapeutic purposes.

## 2. General Protocol for Seed-Mediated Synthesis of GNRs and Their Characterization

**Figure 1:**
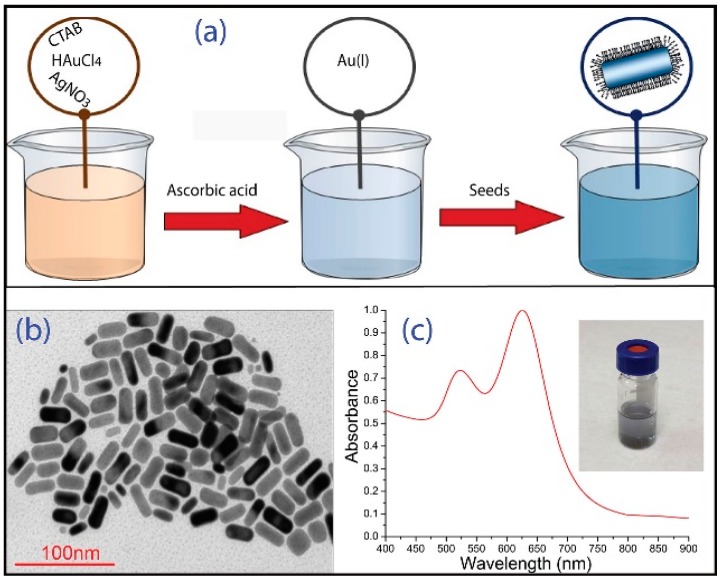
(**a**) Schematization of the synthesis process of gold nanorods (GNRs); (**b**) Transmission electron microscopy (TEM) image of GNRs; and (**c**) Normalized UV-Vis absorption spectrum of an aqueous GNR dispersion (in the inset the picture of a vial containing the same GNR dispersion).

Water-dispersible GNRs were synthesized by means of a seed mediated protocol [19]. The first step involves the preparation of gold “seeds” (GNPs with diameter <3.5 nm) by reducing HAuCl_4_ (5 × 10^−4^ M) with NaBH_4_ (0.01 M) in presence of cetyltrimethylammonium bromide (CTAB) (0.2 M). The seed solution was kept for 2 h under stirring. Meanwhile, the so-called “growth solution” was prepared mixing HAuCl_4_ (0.024 M), CTAB (0.08 M), the proper amount of cyclohexane and acetone, and AgNO_3_ ([Au]/[Ag] = 20). Au(III) was reduced to Au(I) by addiction of ascorbic acid ([ascorbic acid]/[Au^3+^] = 2) obtaining a colorless solution. At this stage, the growth of GNRs was triggered by adding a “seed” solution. The turning of the solution color from colorless to blue indicates the formation of anisotropic particles (Figure 1a). The samples were purified from excess of surfactant by several centrifugation cycles and were optically and morphologically characterized, as shown in Figure 1b,c.

**Figure 2:**
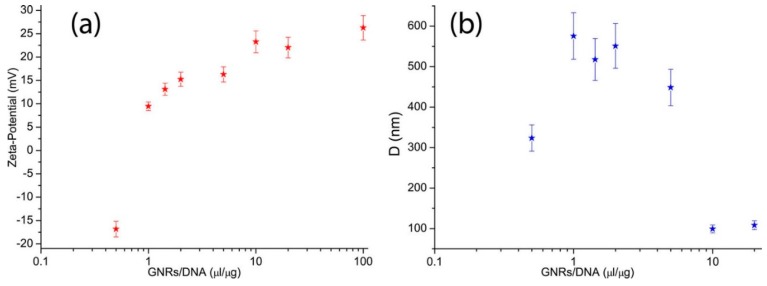
(**a**) ζ-potential and (**b**) hydrodynamic diameter as a function of the GNR/DNA ratio.

Transmission electron microscopy (TEM, by Jeol JEM-1011 microscope, Jeol, Peabody, MA, USA, operating at 100 kV) analyses were performed by depositing a droplet of different aqueous GNR dispersion onto a carbon-coated copper grid, and then allowing the aqueous solvent to evaporate. For a statistical determination of the average GNR size, shape and aspect ratio, at least 200 objects were counted for each investigated sample. The TEM image of Figure 1b indicates that the particle population mainly consists of GNRs having an aspect ratio (AR) of 2.3 ± 0.3. The normalized UV-Vis absorption spectrum of GNRs is characterized by two distinct bands corresponding to the transverse and longitudinal plasmonic oscillation of electrons with incident electromagnetic field at 523 nm and 635 nm, respectively (Figure 2c). The longitudinal Plasmon band of GNRs is known to be very sensitive to AR of GNRs. This effect can be explained by taking into account that the optical properties of GNRs can be predicted in the framework of the Gans theory [20], which describes optical properties of ellipsoidal NPs as based on dipole approximation. Indeed, in aqueous solution the longitudinal LPR wavelength (λ_max_) is linearly proportional to the AR trough the following relationship:

λ_max_= 95AR + 420 (1)

λ_max_ can be modulated in the VIS-NIR spectral range by carefully tuning the numerous experimental parameters involved in the GNRs preparation, such as purity and concentration of reactants, temperature, and specific additives.

## 3. Characterization of GNRs/DNA Complexes

### 3.1. ζ-Potential and Hydrodynamic Diameter Determination

A whole-genome DNA extracted from human blood (concentration ≈ 110 ng/μL), containing approximately 10,000 base pairs (1 bp length ≈ 0.34 nm) and with a contour length of about 3,4 μm was used. For the experiments, GNRs (Concentration = 2.5 × 10^−9^ M) were incubated with DNA at different GNRs/DNA ratios. A first investigation of the electrostatic interaction between positive GNRs and negatively charged DNA molecules was carried out by measuring their ζ-potential and average hydrodynamic diameter, *D*, as a function of the GNR/DNA ratio.

In order to study the effective electric charge of the system, electrophoretic mobility experiments were carried out by means of a laser Doppler electrophoresis technique (Malvern-NanoZetaSizer). The mobility *u* was converted into the ζ-potential using the Smoluchowski relation: ζ-potential = *u*η/ε, where ε is the permittivity of the solvent phase while η is the solvent viscosity. Three independent ζ-potential measurements (called triplicate) were made.. Results are reported as average value ± standard deviation in Figure 2a. For low GNRs concentration (GNRs/DNA = 0.5 µL/µg), complexes exhibit a ζ-potential ≈ −17 mV being DNA molecules negatively charged. Increasing GNR/DNA ratio (0.5 µL/µg < GNRs/DNA < 1 µL/µg), since GNRs are positively charged due to the presence of the CTAB bilayer and DNA is negatively charged, the complex formed by the aggregates undergo a charge inversion effect (the so called isoelectric point) assessed by the change in sign of the ζ-potential value, that from a negative value due to the excess of DNA, become positive due to the excess of GNRs. Further increase in the GNRs/DNA ratio (>1 µL/µg) induces the formation of positively charged complexes with a final ζ-potential ≈ 25 mV. We have also monitored the hydrodynamic diameter (*D*) as a function of the GNR/DNA ratio by performing Dynamic Light Scattering (DLS) experiments (Figure 2b). Measurements have been performed at 37 °C by means of a Malvern NanoZetaSizer spectrometer equipped with a 5 mW HeNe laser (λ = 632.8 nm) and a digital logarithmic correlator. Scattered light is collected at θ = 173°, the angle between incident radiation and scattered light wave vectors. The correlation between the scattered light and *D* has been reported in details elsewhere [21]. For GNRs/DNA = 0.5 µL/µg, the complexes are about 300 nm in size while by increasing the GNRs/DNA ratio, size of the complex gradually increased until reaching a maximum for 0.5 µL/µg < GNRs/DNA < 1 µL/µg. Indeed, for positively or negatively charged complexes the electrostatic charges create a repulsive barrier, which dominates over short-range attractive van der Waals forces and prevents aggregation. However, around the isoelectric point (0.5 µL/µg < GNRs/DNA < 1 µL/µg), anionic (DNA) and cationic (GNRs) charges neutralized each other (charge inversion effect, Figure 2a). As a result, the attractive van der Waals interactions allow aggregates to come into contact leading to large size GNRs/DNA complexes (*D* ≈ 600 nm). 

Further increase in the GNRs/DNA ratio causes the formation of positively charged, decreasing-size (*D* ≈ 100 nm) complexes (reentrant condensation). *Note: the reentrant condensation is a phenomenon that occurs when the size of the agglomerates* *continuously increases reaching a maximum at a well-defined value and then decreases*. Therefore, in view of potential applications, the complex with GNRs/DNA = 10 represents the most promising candidate for genomic DNA delivery. Indeed, this ratio ensures positive ζ-potential that is necessary for efficient interaction with the plasma membrane of target cells that contains cell surface proteoglycans with negatively charged sulfate groups on the extracellular side [22] and prevent formation of large size complexes. *Note:* *Proteoglycans consist of a core protein and one or more covalently attached polysaccharide chains (glycosaminoglycan chains).* Indeed, at GNR/DNA = 10, complexes are small in size (100 nm). It is widely accepted that complexes with sizes smaller than 200 nm can avoid recognition by the immune system, thus prolonging circulation time in the blood. In addition, once at the target site, they can be efficiently internalized within target cells [23].

### 3.2. Electrophoresis

A preliminary investigation of the interaction between GNRs and DNA was carried out by performing a gel electrophoresis analysis, a method useful for separation and, sometimes, purification purposed of macromolecules, especially proteins and nucleic acids, that differ in size, charge or conformation. When charged molecules are in an electric field, they migrate toward either the positive or negative pole according to their charge through an agarose matrix [24]. In this way, during their electrophoretic run, the macromolecules can be separated according to their length since the gel matrix allows shorter DNA fragments to migrate faster than larger ones. The mixture of DNA and GNRs has been then added of a small amount (1% in weight) of a positively charged blue dye (ethidium bromide, EtBr), which is an intercalating agent resembling a DNA base pair [25]. Due to such a unique structure, it can easily intercalate into DNA strand. EtBr has UV absorbance maxima at 360 nm and an emission maximum at 590 nm, with its orange fluorescence increasing of about 20 times upon binding to DNA.

**Figure 3:**
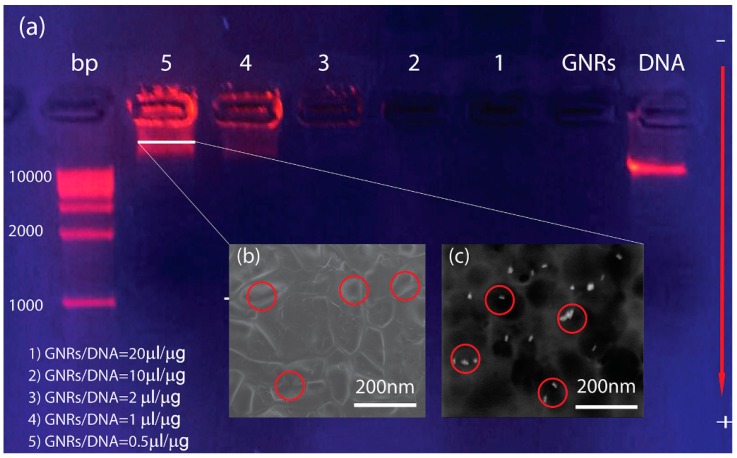
Gel electrophoresis (**a**) along with the environmental scanning electron microscope (ESEM) cross section of the line 5 of agarose matrix before (without the GNRs/DNA solution) (**b**) and after (with the GNRs/DNA solution) the electrophoretic run (**c**).

Subsequently, the obtained solutions have been pipetted in the agarose matrix molded within wells (Figure 3a), containing also DNA filaments of known size distribution (in the first well on the left of Figure 3a), which have been used as markers. The agarose matrix has been then placed on a UV lamp and covered with a UV glass filter. After the power supply has turned on, the DC voltage (120 V, 0.78 mA) induced a migration of the pure DNA (Figure 3a, first well on the right) towards the positive electrode. Observation of a single fluorescent band indicates that DNA molecules have almost the same length, which includes approximately 10,000 base pairs. Figure 3a (second well on the right) shows that the GNRs dispersion has been not affected by the electrophoretic run since GNRs are positively charged due to the presence of the CTAB bilayer. Indeed, GNRs do not exhibit any visible fluorescence band in the well since no binding event can be thought to occur between GNRs and EtBr and, therefore, the water-based GNRs dispersion represent an effective hydrophobic environment able to induce a strong quenching of the EtBr fluorescence. Different values of GNRs/DNA ratios (displayed in Figure 3a) have been monitored in order to study the complexation of DNA with GNRs in different experimental conditions such as positive, neutral and negative effective electric charge of the system. For high GNRs concentration (10 µL/µg < GNRs/DNA <20 µL/µg) the average ζ-potential is positive (see Figure 2a), but no fluorescence band is visible; most likely this is due to the high concentration of GNRs, which can induce, once again, a strong quenching of the EtBr fluorescence. For GNRs/DNA = 3, the average ζ-potential is still positive (see Figure 2a) but a visible fluorescence band appears very close to the well, pointing out a very slow and hindered migration toward the positive electrode. For low GNRs concentration (0.5 µL/µg < GNRs/DNA < 1 µL/µg), the average ζ-potential is negative and the fluorescence appears split and comes from two main sites: a first one, closer to the well, reasonably ascribable to the electrostatic conjugation between GNRs and DNA, and a second one, at about a 10,000 base pairs distance. This evidence can be reasonably explained by thinking that, under these conditions, electrostatic interaction between GNRs and DNA are saturated and therefore all the non-conjugated DNA molecules or even the over conjugated GNRs/DNA complexes (means complexes with an effective negative electric charge) are free to run towards the positive electrode. In order to verify this hypothesis, we have performed an environmental scanning electron microscope (ESEM) characterization by analyzing the cross section (across well 5 between the two most intense sites) of the agarose matrix before (without the GNRs/DNA solution) and after (with the GNRs/DNA solution) the electrophoretic run. Figure 3b shows the roughly circular porous structure (average pores size ≈ 120 nm) of the empty agarose matrix while Figure 3c shows the same image after the electrophoretic run. As evidence, the presence of GNRs is well visible and they are distributed around the pores of the agarose matrix (note: there is no evidence of DNA molecules since, due to the high energy of the impinging electron beam, the DNA can easily evaporate). This result confirms that also the over conjugated GNRs/DNA complexes are free to run towards the positive electrode as hypothesized earlier.

## 4. Photo-Thermal Characterization

**Figure 4:**
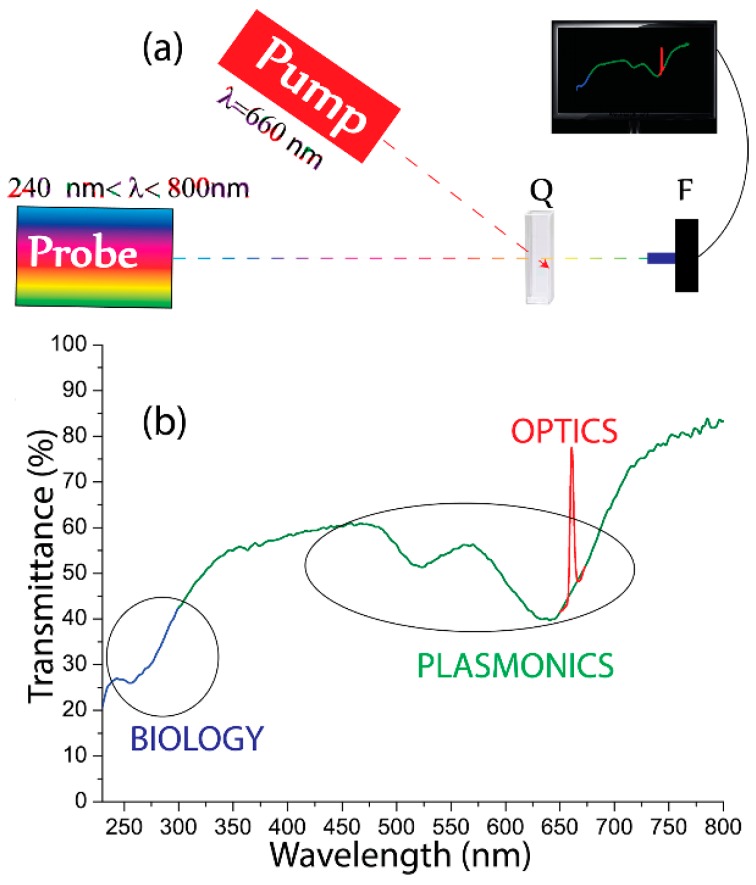
(**a**) All-optical setup for sample characterization (*Q* is the quartz cuvette and *F* is the transmission optical fiber); (**b**) Transmission spectrum of the GNRs/DNA solution under optical illumination.

Experiments devoted to investigate the influence, on the GNRs/DNA complexes, of the local heating induced by a suitable optical radiation trough the GNRs resonance, have been performed by using the all-optical setup reported in Figure 4a. A collimated white source (240 nm < λ < 800 nm) has been used to monitor spectral properties of the GNRs /DNA solution, along with a CW pump laser emitting at λ = 690 nm (*P*_pump_ = 0.14 W/cm^2^), in the high absorption range (longitudinal band) of the GNRs. For sake of simplicity, an unfocused pump beam, impinging on the sample with an oval cross section of about (3 × 4) mm^2^ has been used. The sample is placed on a hot plate for control experiments based on delocalized/uniform heat.

The photo-heating experiments were carried out on the solution with GNRs/DNA = 10, that has been identified it as the most promising candidate for genomic DNA delivery. Indeed, the same sample exhibits excellent photo-thermal sensitivity due to the high concentration of GNRs. Figure 4b shows the transmission spectrum of the sample. It is worth pointing out the presence of three distinctive features highlighted with different colors: (i) DNA absorption peak (named Biology) at λ = 260 nm (Figure 4b, blue color) since the DNA solution exhibits a well-known sharp maximum at 260 nm due to the absorption of the subunits of nucleic acids (purines and pyrimidines) [26]; (ii) GNRs absorption (named Plasmonics), namely transverse and longitudinal LPRs, at 520 nm and 660 nm, respectively (Figure 4b, green color); and (iii) a monochromatic narrow peak (named Optics) due to the presence of the external pump beam at λ = 690 nm (Figure 4b, red color).

**Figure 5:**
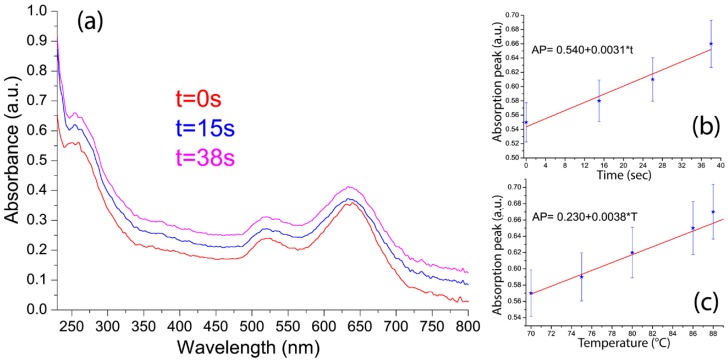
Absorption spectra of the sample for different values of the (**a**) illumination time; linear fit of the intensity of the DNA absorption peak (at λ = 260 nm) *versus* the (**b**) illumination time and (**c**) temperature.

By optically pumping the same probed sample area, the photoexcitation of GNRs induces an electric-driven Joule heating with a consequent energy exchange with the surrounding medium, namely DNA here [27,28]. This local heating induces a gradually DNA melting and, concomitantly, the double-stranded genomic DNA unwinds and separates into single-stranded DNA through the breaking of hydrophobic stacking attractions between the base pairs. As a consequence (Figure 5a, from red to magenta curve), the absorbance at 260 nm rises (this effect being known as a hyperchromic effect). Interestingly, the plot of the intensity of the DNA absorption peak *versus* the illumination time exhibits a linear behavior (Figure 5b, measurements have been performed in triplicate and results are reported as average value ± standard deviation) while no remarkable shift has been observed for the transverse and longitudinal band of GNRs due to the low change of the refractive index variation of the surrounding medium (DNA in our case). A further validation of the GNRs induced local heating on the DNA, has been achieved by performing a control experiment, namely by varying the sample temperature from 70 °C up to 90 °C and monitoring the absorption spectrum (Figure 5c). The delocalized heating has been found, again, to induce a gradual DNA melting with a consequent enhancement of the absorption peak at 260 nm, thus confirming that the photo-thermal nature of the behavior reported in Figure 5b. The two calibration functions reported in Figure 5b,c (absorption peak, AP *vs.* time and AP *vs.* temperature) exhibit the same linear behavior allowing the prediction of the temperature at a given illumination time through the equation:

(2) $$T=\frac{\left(AP-0.230\right)}{0.0038}$$

A sensitivity of 0.06 °C can be achieved using this method, thus providing a direct, fast and precise approach to measuring local temperatures in a biocompatible environment by exploiting the DNA melting process. It is worth mentioning that the reported method has been investigated in a temperature range between 70 and 90 °C in order to study the photo-induced melting properties of the DNA solution. However, the same approach can be extended to other, lower, temperature range (e.g., 25–70 °C), by using a different calibration curve since the full melting diagram of a DNA solution exhibits a sigmoidal behavior, due to superimposition of two linear calibration curves.

## 5. Conclusions

We have reported on the characterization of the electrostatic interaction between GNRs dispersion and a DNA solution. The study shows that by carefully controlling the GNR/DNA ratio, aggregates formed by the two moieties undergo a charge inversion effect, that allow to distinguish negatively and positively charged complexes. These results along with the results of the dynamic light scattering experiments evidence that at GNR/DNA = 10 complexes are small in size (100 nm) and positively charged. As a consequence, this particular GNR/DNA ratio has been identified as the best candidate for genomic DNA delivery. Gel electrophoresis results combined with an environmental scanning electron microscopy data confirm the complex behavior, pointing out that the over conjugated GNRs/DNA complexes (means complexes with an effective negative electric charge) are free to run through the agarose matrix. Localized heating experiments realized by using a bio-transparent optical radiation (λ = 690 nm) combined with uniform heating (from 70 °C up to 90 °C) demonstrated that the system represent an accurate temperature sensor able to monitor heating variation by means of the DNA melting process (at λ = 260 nm) with a sensitivity of about 0.06 °C. Overall, our results represent, not only a general understanding of the fundamental interaction between nano and bio materials [29], but depict an important step-forward towards the realization of plasmonic-assisted therapeutics.
